# Regulatory B Cells Are Decreased and Impaired in Their Function in Peripheral Maternal Blood in Pre-term Birth

**DOI:** 10.3389/fimmu.2020.00386

**Published:** 2020-03-20

**Authors:** Mandy Busse, Kim-Norina Jutta Campe, Anke Redlich, Anika Oettel, Roland Hartig, Serban-Dan Costa, Ana Claudia Zenclussen

**Affiliations:** ^1^Experimental Obstetrics and Gynecology, Medical Faculty, Otto-von-Guericke University, Magdeburg, Germany; ^2^University Hospital for Gynecology, Obstetrics, and Reproductive Medicine, Otto-von-Guericke University, Magdeburg, Germany; ^3^Medical Faculty, Institute for Molecular and Clinical Immunology, Otto-von-Guericke University, Magdeburg, Germany

**Keywords:** preterm birth, B cells, pregnancy, interleukin-6, regulatory B cells

## Abstract

Preterm birth (PTB) is defined as birth before 37 completed weeks of gestation. The causes of PTB are multiple and complex, the underlying pathophysiology being largely unknown. Interferences in the fine-tuned balance of the maternal immune system have been pointed to as one possible cause of PTB. Regulatory B cells (Breg) are part of the adaptive immune response, and recent data suggest that they may contribute to a healthy pregnancy by their regulatory/suppressive function.

We investigated the frequency of Breg cells in peripheral blood of women undergoing PTB and control women immediately before giving birth via cesarean section. We detected an enhanced number of B cells, but a reduced number of Breg cells in women delivering preterm. In addition, the percentage of IL-10-producing B cells was decreased in PTB following stimulation with TLR agonists CpG or LPS, alone or combined with CD40L. This was associated with increased levels of pro-inflammatory cytokines in maternal serum. Moreover, isolated maternal B cells before delivering premature babies secreted higher level of the pro-inflammatory cytokine IL-6. No alterations in the frequency of regulatory T cells were found.

Our data indicate that alterations in the number and function of Breg cells in peripheral maternal blood contribute to the immunological changes observed in preterm delivery and suggest these cells as important regulators of maternal immune responses.

## Introduction

Preterm birth (PTB) is defined as the delivery of a baby before 37 completed weeks of gestation. PTB is still a leading course for neonatal morbidity and mortality (1). Worldwide, about 15 million babies and their mothers are affected every year with stable or even increasing frequencies. The PTB rate in Germany is one of the highest across Europe (2, 3).

PTB is a syndrome and has multiple etiologies. About one third of all PTBs in high-income countries are medically indicated/iatrogenic; the remaining approximately 70% are spontaneous (4). PTB will be medically induced when the risk for the fetus or the mother outweighs the benefit to continue pregnancy, for example, in conditions such as preeclampsia or intrauterine growth restriction (IUGR). Preeclampsia is a pregnancy-associated disorder characterized by hypertension and a variety of organ failures (5), while IUGR is defined as pathologic inhibition of intrauterine fetal growth and the failure of the fetus to achieve its growth potential (6).

Spontaneous PTB might be the consequence of preterm labor with cervical dilation or preterm premature rupture of membranes (PPROM). Risk factors include uteroplacental ischemia and hemorrhage, a shortened cervical length and polyhydramnios (7). Infection significantly contributes to PTB; the most frequent route of intrauterine infection is ascension from the vagina. Several studies suggested that intrauterine infection accounts for ~30% of PTBs, but this might represent a minimum due to difficulties in the detection of invaded microorganisms via conventional culture techniques (8). In addition, the frequency increases with decrease of gestational age at PTB.

Invading pathogens are recognized by pattern-recognition receptors such as toll-like receptors (TLR) by cells primarily of the innate immune system, which react by releasing inflammatory mediators, among them interleukin (IL)-6, IL-1β, IL-8, and tumor necrosis factor (TNF)-α. Endotoxins like lipopolysaccharide (LPS) and inflammatory cytokines stimulate the production of prostaglandins, more inflammatory mediators and matrix-degrading enzymes. While prostaglandins stimulate uterine contractility, the degradation of the extracellular matrix in the fetal membranes might result in PPROM. Infections might also contribute to approximately 5–15% of IUGR (9).

It is key to understand the immune response at different stages of pregnancy and term labor, but also in PTB, to develop new therapeutic concepts to identify and treat patients at risk. Throughout pregnancy, maternal–fetal tolerance is established by a shift from inflammatory toward antigen-specific anti-inflammatory immunity. However, parturition is an inflammatory process; therefore another shift in the immune response must be implemented at the end of pregnancy. Among the cells of the adaptive immune system, B cells need to be studied in more detail and depth. B cells contribute to a healthy pregnancy via several mechanisms: they produce asymmetric antibodies (10), and particularly regulatory B (Breg) cells contribute to establish and maintain maternal-fetal tolerance (11). Breg cells might employ several mechanisms for tolerance, maintenance and immune homeostasis, the most important one by the secretion of IL-10 (11). Multiple human B cell subsets have shown an ability produce IL-10, among them CD24^hi^CD38^hi^ B cells (12, 13), CD24^hi^CD27+ B10 cells (14), and CD1d^hi^CD5+ B cells (15). Our group has also identified plasma cell alloantigen 1 (PC1) as a marker for murine IL-10-producing B cells (16). These B cell populations do not only differ in their phenotype, but also in their functional activities. The frequency of Breg cells is below 10% of B cells in peripheral blood (13), and this proportion is altered in pregnancy (17). Despite this, B cells also act as professional antigen-presenting cells (APC). They express co-stimulatory molecules such as CD40, CD80, and CD86 and TLRs, which enable them to respond to microbial components such as LPS or CpG. Consequently, they might differentiate either into immunoregulatory Breg cells (14) or into B cells secreting pro-inflammatory mediators. In both cases, they might influence other immune cells and enhance or dampen the upcoming inflammatory immune response. Therefore, B cells emerge as candidates with the capacity of considerably interfering with the established maternal–fetal tolerance in the event of an infection or inflammation. This might be counter-regulated by enhancing Breg numbers among the B cell pool but may also lead to PTB if pro-inflammatory B cells among B cells are overwhelming. This may also depend on the extent of the inflammatory insult.

The aim of our study was to analyze the frequency and function of B cells, in particular Breg cells, at term or in the event of PTB. We hypothesized that a dysregulated balance between pro- and anti-inflammatory B cells might contribute to the immunological alterations seen in PTB. We found that the number and function of B cells and Breg cells is disturbed in maternal blood immediately before the onset of PTB, further establishing an important role for B cells in a healthy pregnancy.

## Materials and Methods

### Human Subjects

The study was approved by the ethics committee of the Otto-von-Guericke University medical faculty (EK28/08). Patients who underwent a planned cesarean section either at term (>37 completed weeks of gestation) or preterm (<37 completed weeks of gestation) were properly informed about the purpose of the study (“Investigation of B cells in maternal blood in term and preterm birth”) and gave written consent before participating. Patients included in this study were recruited between April 2016 and February 2017. The demographic data of the patients are summarized in Table 1. Up to 40 ml venous EDTA blood were taken from each pregnant woman and immediately stored on ice. Blood was processed within 1 h.

**Table 1:** Study cohort.

	**TD**	**PTB**	
**Characteristics**	***N*** **=** **10**	***N*** **=** **8**	***p***
**MATERNAL**			
Age	31.40 ± 5.99	31.88 ± 4.52	0.8550
GA (weeks)	39.50 ± 1.58	32.00 ± 3.46	<0.0001
Pregnancy	2.6 ± 1.27	3.88 ± 2.85	0.2210
Parity	2.3 ± 1.25	2.5 ± 1.41	0.7545
**NEONATAL**			
birth weight (g)	3308 ± 556	1650 ± 567	<0.0001
APGAR 1 min.	8.9 ± 0.57	8.4 ± 0.53	0.1158
APGAR 5 min.	9.5 ± 0.97	9.4 ± 0.79	0.7311
APGAR 10 min.	9.8 ± 0.63	9.6 ± 0.53	0.3527
pH (cord blood)	7.32 ± 0.036	7.38 ± 0.033	0.0053
base excess	−1.38 ± 1.784	−1.31 ± 0.855	0.9174

*Ten women delivering term (term delivery, TD) and eight women delivering preterm (preterm birth, PTB) were included in the study. Maternal characteristics included age of the mother, gestational age (GA; weeks), number of pregnancies, and parities. Neonatal features included birth weight [grams (g)], APGAR scores at 1, 5, and 10 min after birth, the cord blood pH value and base excess*.

### Stimulation of PBMCs

PBMCs were separated by Ficoll-density gradient centrifugation and cultured in RPMI 1640 supplemented with 1% penicillin/streptomycin and 10% fetal bovine serum (FBS). 2 × 10^6^ PBMCs/ml were stimulated with PMA (50 ng/ml) and ionomycin (500 ng/ml; both Sigma Aldrich, Darmstadt, Germany) alone or combined with LPS (10 μg/ml) or CpG ODN2006 (10 μg/ml) for 5 h at 37°C and 5% CO_2_. Brefeldin A (Biolegend; San Diego, USA) was added to all wells, including medium control.

2 × 10^6^ PBMCs/ml were stimulated with LPS (*E. coli* serotype 0111:B4; 10 μg/ml; Sigma Aldrich, Darmstadt, Germany) or CpG ODN2006 (10 μg/ml; Invivogen; San Diego, USA) alone or combined with human CD40L (1 μg/ml; R&D systems; Minneapolis, USA) for 48 h at 37°C and 5% CO_2_. PMA (50 ng/ml), ionomycin (500 ng/ml) and Brefeldin A was added for the last 5 h.

### Isolation and Stimulation of B Cells

B cells were isolated using the human B cell isolation kit II (Miltenyi Biotech, Bergisch Gladbach, Germany). Isolated B cells were stimulated with LPS (10 μg/ml) or CpG ODN2006 (10 μg/ml) alone or combined with human CD40L (1 μg/ml) for either 24 h (for co-culture experiments) or 72 h (for recovery of supernatants) at 37°C and 5% CO_2_.

### Cell Staining and Flow Cytometry

3 × 10^5^ PBMCs were stained for cell surface markers for 30 min at 4°C. The following anti-human antibodies were used: FITC-labeled CD19 (clone HIB19), APC-labeled CD24 (clone eBioSN3), PE-Cy7-labeled CD38 (clone HB7), PE-labeled CD5 (clone UCHT2), and APC-labeled CD1d (clone 51.1). To analyze the intracellular expression of IL-10, cells were fixed for 30 min with Fix and Perm and stained with PerCP-Cy5.5-labeled IL-10 (clone JES3-9D7; all reagents ebioscience, San Diego, USA).

T cells were analyzed as follows: FITC-labeled CD4 (clone RPA-T4) and PerCP-Cy5.5-labeled CD25 (clone BC96) were stained at the cell surface. Following a fixation for 30 min with Fix and Perm, the intracellular staining of APC-labeled Foxp3 (clone 236A/E7) was performed for 30 min at 4°C. To ensure correct gating of rare cell populations, we used Fluorescence Minus One (FMO) controls for each antibody (18). Measurements were performed with LSR Fortessa (BD Biosciences, Heidelberg, Germany) and analyzed with FloJo software (Ashland, Oregon, USA).

### Cytokine Detection in Plasma Samples and Supernatants

Cytokines were quantified by the cytometric bead array (CBA) human Th1/Th2/Th17 Cytokine Kit from Biolegend (San Diego, USA) following supplier's recommendation.

### Data Analysis and Statistics

Statistical analysis was performed using GraphPad Prism 8.0 software. Normality of distribution was determined by Shapiro-Wilk test. Data were analyzed by either Mann-Whitney-U test or two-way ANOVA, then followed by either Bonferroni's or Sidak's multiple comparison test.

## Results

### Study Cohort

Ten women undergoing cesarean section at term [term delivery, TD; mean gestational age (GA) = 39.5 weeks] and eight women delivering preterm (preterm birth, PTB), also via cesarean section (mean GA = 32.0 weeks; *p* < 0.0001), participated in the study (Table 1). Between the two groups, there were no differences in maternal age, pregnancy numbers, parity numbers, APGAR scores or cord blood base excess. Babies born preterm had a decreased birth weight (mean = 1,650 g compared to mean = 3,308 g in TD; meaning a reduction of 49.9% to TD; *p* < 0.0001) and a higher cord blood pH value (mean = 7.38 compared to mean = 7.32 in TD; *p* = 0.0053). Reasons for preterm birth in our patient cohort included IUGR, PPROM, amniotic infection or polyhydramnios. Supplementary Table 1 depicts the CRP levels and leukocytes numbers at the first day of lung maturation treatment (time point (TP) 1) and on the day of delivery, 2–3 days after TP1. The samples used for CRP and leukocyte numbers are the same samples used for our *in vitro* investigations (usually within 30 min before delivery). Blood from women who delivered at term was also taken within 30 min before delivery. We detected no difference in the leukocyte numbers within the PTB and TD groups immediately before delivery (Supplementary Figure 2). Reasons to deliver via cesarean section (C-section) included previous C-sections and breech.

### PTB Is Associated With an Enhanced Level of Pro-inflammatory Cytokines in Maternal Blood

**Figure 1:**
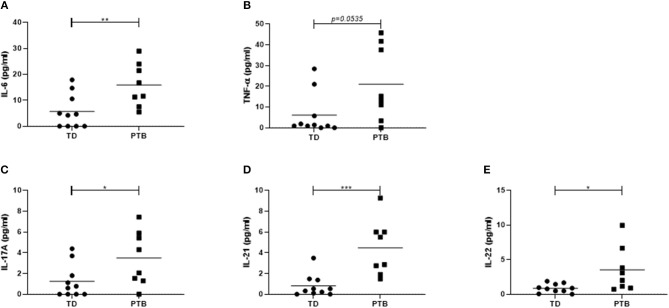
Cytokine level in maternal plasma. Maternal plasma was taken immediately before delivery. Cytokine levels were determined using a Th panel multiplex bead-based assay. The levels of IL-6 **(A)**, TNF-α **(B)**, IL-17A **(C)**, IL-21 **(D)**, and IL-22 **(E)** were presented. Shown are the individual values and the mean. Data were analyzed by Mann-Whitney test; *p < 0.05; **p < 0.005; ***p < 0.001.

First, we investigated the cytokine level in the maternal peripheral blood obtained immediate before delivering. In PTB patients, we found increased plasma level of IL-6 (*p* = 0.0079; 2.8 times higher in PTB than in TD), TNF-α (*p* = 0.0535; 3.1 times higher in PTB than in TD), and IL-17A (*p* = 0.0413; 2.7 times higher in PTB than in TD). Also, the level of IL-21 (p = 0.0007; 5 times higher in PTB than in TD) and IL-22 (*p* = 0.0149; 4.1 times higher in PTB than in TD) were enhanced in plasma of PTB patients compared to TD, all shown in Figure 1.

### Dysregulated Breg Cell Numbers in PTB

**Figure 2:**
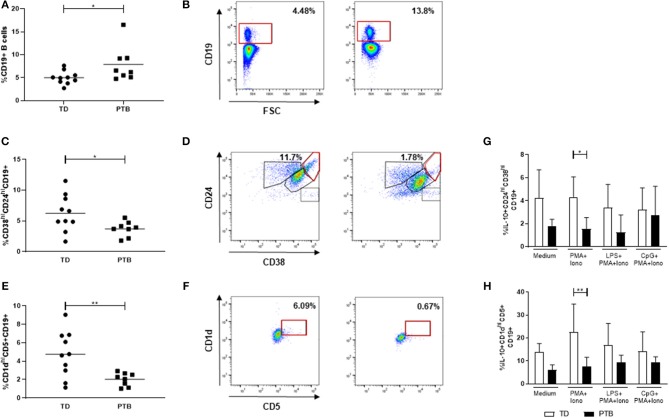
Regulatory B cell populations in maternal blood before delivery. The percentage of CD19+ B cells **(A)**, CD38^hi^CD24^hi^CD19+ Breg cells **(C)** and CD1d^hi^CD5+CD19+ Breg cells **(E)** and the corresponding gating strategies **(B,D,F)** were calculated within maternal blood obtained immediate before delivering. Data were analyzed by Mann-Whitney test. Shown is the mean value. PBMCs were stimulated for 5 h in the presence of Brefeldin A alone or combined with PMA and ionomycin, partly added by LPS or CpG. Intracellular IL-10 secretion within the above-mentioned Breg populations was determined by flow cytometry. Data were analyzed using two-way ANOVA with Bonferroni's multiple comparisons test, shown is the mean and SD. TD, term delivery; PTB, preterm birth; *p < 0.05; **p < 0.005.

We determined the frequency of CD19+ B cells and two separate Breg populations, characterized by the expression of either CD24 and CD38 or CD1d and CD5 (gating strategy shown in Figures 2B,D,F). While the number of total B cells in maternal blood of PTB patients was enhanced (*p* = 0.0343; Figure 2A), the number of both Breg populations, CD38^hi^CD24^hi^CD19+ Breg cells (*p* = 0.0434; Figure 2C), and CD1d^hi^CD5+CD19+ Breg cells (*p* = 0.0085) was decreased (Figure 2E).

Next, we determined the frequency of IL-10-producing B cells. Therefore, we stimulated the isolated PBMCs with PMA and ionomycin, partly added by the TLR agonists LPS or CpG, in the presence of Brefeldin A, which was also added to the medium control, according to Iwata et al. (14). Stimulation of PBMCs with PMA and ionomycin induced the expression of IL-10 in CD38^hi^CD24^hi^CD19+ (Figure 2G) and CD1d^hi^CD5+CD19+ Breg cells (Figure 2H) in women delivering at term compared to women delivering preterm. This trend was detectable in all culture conditions but did not reach statistical significance. The addition of LPS or CpG did not further enhance the IL-10 production by Breg cells.

Neither the number of CD4+ T cells (Supplementary Figures 1A,B) nor the frequency of regulatory T cells (Treg; Supplementary Figures 1C,D) was altered in patients delivering preterm compared to women giving birth at term.

### Maturation of Breg Cell Populations Is Disturbed in PTB

**Figure 3:**
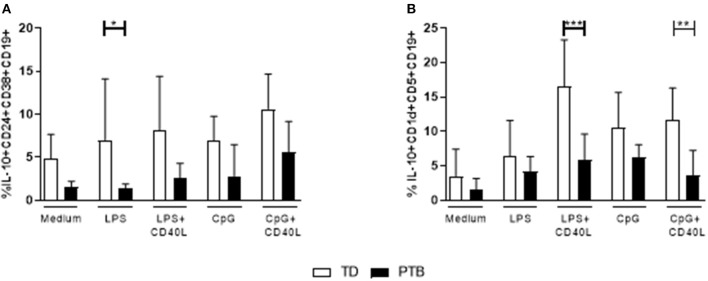
Regulatory B cell populations in maternal blood before delivery. The frequency of IL-10-producing CD38^hi^CD24^hi^CD19+ **(A)** and CD1d^hi^CD5+CD19+ Breg cells **(B)** was determined following stimulation of PBMCs with LPS or CpG alone or combined with CD40L for 48 h. For the last 5 h, Brefeldin A PMA and ionomycin was added to the cultures. Breg cells were detected by flow cytometry. Data were analyzed using two-way ANOVA with Sidak's multiple comparisons test. Shown is the mean and SD. TD, term delivery; PTB, preterm birth; *p < 0.05; **p < 0.005; ***p < 0.001.

The ability of B cells to mature into Breg cells was investigated according to Iwata et al. (14) by stimulating PBMCs for 48 h with LPS or CpG, either alone or combined with CD40L. Control cells were cultured in medium. Brefeldin A, PMA, and ionomycin were added for the last 5 h. IL-10 production within Breg populations was detected by flow cytometry. We found that the secretion of IL-10 was enhanced in LPS-stimulated CD38^hi^CD24^hi^CD19+ Breg cells from women delivering at term compared to patients delivering preterm (Figure 3A). Women delivering preterm also showed a diminished IL-10 release by CD1d^hi^CD5+CD19+ Breg cells following stimulation of PBMCs with either LPS or CpG in the presence of CD40L (Figure 3B). Again, this trend was determined in all culturing conditions but did not gain significance. Representative pictures obtained by flow cytometry were shown in Supplementary Figure 3, the corresponding FMO controls in Supplementary Figure 4.

### B Cells From PTB Patients Secreted Pro-inflammatory Cytokines

**Figure 4:**
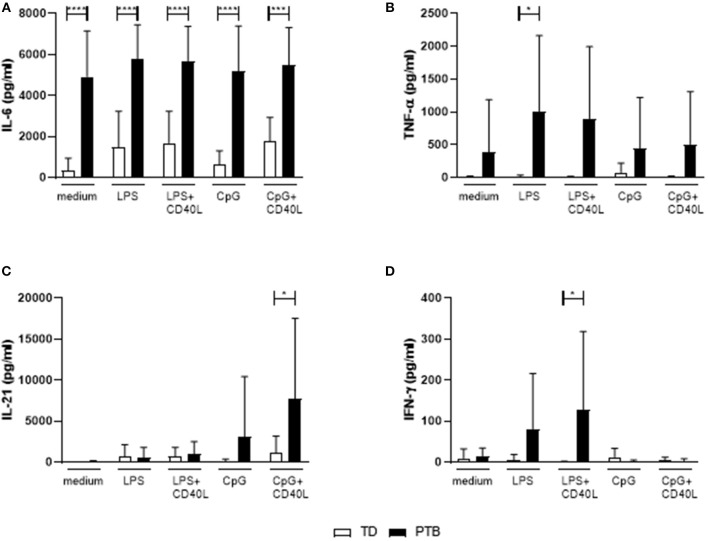
Secretion of cytokines by maternal peripheral blood B cells immediate before delivery. Isolated maternal B cells were stimulated with LPS or CpG alone or combined with CD40L for 72 h. Supernatant was harvested, and the cytokine level was determined using a Th panel multiplex bead-based assay. Presented are the levels of IL-6 **(A)**, TNF-α **(B)**, IL-21 **(C)**, and IFN-γ **(D)**. Shown are the mean values and the SD. Data were analyzed by two-way ANOVA with Sidak's multiple comparisons test; *p < 0.05; ***p < 0.001; ****p < 0.0001.

The level of cytokines produced by B cells without the presence of other immune cells was determined by stimulating isolated B cells for 72 h with LPS or CpG, either alone or combined with CD40L. Released cytokines in the supernatant were analyzed by a Th cytokine multiplex assay using flow cytometry. We found that B cells isolated from PTB patients had a strongly enhanced ability to secrete IL-6, even with control medium (Figure 4A). The production of IL-6 by unstimulated B cells in PTB correlated with the gestational week (Supplementary Figure 5). In addition, other pro-inflammatory cytokines were produced by B cells from PTB women to a higher amount under various culture conditions: TNF-α was significantly enhanced following LPS stimulation (Figure 4B), IL-21 after CpG+CD40L (Figure 4C) and IFN-γ following LPS+CD40L stimulation (Figure 4D). All other analyzed cytokines, among them IL-10, did not present significant differences (data not shown).

## Discussion

The immense challenge in unraveling PTB etiology is to work out the extremely complex interactions between gestational hormones, components of the immune system, and reproductive tissues. The role of B cells in these interactions is only poorly understood.

We have recently shown that IL-10 and IL-10-producing Breg cells are important for fetal survival in a LPS-mediated mouse model of intrauterine fetal death (11). The importance of IL-10 was also proven in murine LPS-induced preterm labor (19). It was previously reported that PTB was associated with IL-10 gene polymorphisms and decreased IL-10 mRNA (20). Independent of the presence of a bacterial vaginosis, decreased IL-10 levels at mid-pregnancy were suggested to be useful as an early predictor of PTB (21). Moreover, stimulation of maternal PBMCs from women with recurrent PTB, PPROM, preeclampsia, or IUGR resulted in lower levels of IL-10 compared to gestational age-matched, healthy pregnant women (22, 23). PTB is often associated with enhanced pro-inflammatory mediators, such as IL-6 and TNF-α (24). Given the fact that B cells are able to produce both pro- and anti-inflammatory mediators, they can incline the balance to a healthy pregnancy but also to pregnancy complications depending on the situation and micromilieu.

In our study, we first determined the number of CD19+ B cells in maternal blood and found an enhanced frequency in women immediately before delivering preterm. During healthy pregnancy, several studies had shown that the absolute counts as well as the frequency of B cells were decreased (25), above all in the third trimester (17, 26, 27). In PTB, the number of CD19+ B cells in the peripheral blood of patients was described to be higher compared to controls (28). Since the frequency of total CD19+ B cells was increased but the percentage of Breg cells decreased, other B cell populations must be responsible for this effect. As we detected enhanced level of inflammatory cytokines in maternal plasma and a strong release of IL-6 by unstimulated B cells in PTB, it is likely that these cells are responsible for the increased B cell numbers. The end effect would be a shift of B cells from immunoregulatory toward inflammatory subtypes.

It has to be taken into account that PTB patients received corticosteroids (Betamethasone) to induce lung maturation of their babies. This treatment may alter the number of leukocytes; it is reported to increase the number of granulocytes and decrease the lymphocyte number (28–30). This effect may last for 2–3 days, and in this study, it does not seem to influence our results since we did not observe differences in the leukocyte count between TD and PTB patients immediately before delivery.

Beside their function as producer of normal and asymmetric antibodies and professional antigen-presenting cells, B cells also exert immunoregulatory functions and therefore contribute to maternal-fetal tolerance. In humans, Breg cells are characterized by the high expression of CD24 and CD38. Their number is increased after pregnancy onset compared to non-pregnant individuals (31) and decreases again close to term (17, 32). The reason for the decrease in Breg cell number might be, at least in part, the increase in inflammatory, parturition-preparing mediators. While this is a normal process, taking place at the end of pregnancy, these events might be triggered much earlier in PTB. We found here that the frequency of these cells is even lower than at term in the samples from women undergoing PTB.

Besides their established phenotype in mice, the molecules CD5 and CD1d might also define a human Breg population (15, 33, 34). It is important to separate these cells from CD5-expressing B1a B cells. This B cell population produces natural antibodies and might also be involved in autoimmune disorders (35, 36). In preeclampsia, we have showed that CD5+CD19+ B cells might serve as indicators for this pregnancy-related disorder, at least partly by the release of autoantibodies connected with disorder-specific symptoms (37). In the present study, we report that women delivering preterm had a decreased percentage of CD1d^hi^CD5+CD19+ B cells and that their IL-10 producing CD1d^hi^CD5+CD19+ B cell frequency was also diminished. Decreased absolute numbers and frequencies of circulating CD5+ B cells were already reported in previous studies in healthy pregnant women (25, 38), but no information about the concomitant expression of CD1d was delivered.

It is tempting to speculate that the reduction in Breg cell frequency and the diminished ability to produce IL-10 will disturb the maternal-fetal tolerance and result in the induction of labor. Most of our patients suffered from PPROM. There are multiple causes for PPROM, including systemic (environmental, nutritional, and genetic) factors, uterine, fetal, and uteroplacental causes (39, 40). Infections, proven or undetected, significantly contribute to the pathology. Intraamniotic infection or inflammation enhanced the number of B cells in the amniotic fluid (41). PTB might also be associated with chronic deciduitis, characterized by extensive lymphocyte infiltration and the presence of plasma cells in the placenta, particularly in the basal plate (42, 43). Gomez-Lopez et al. reported that during labor, the amnion is responsible for chemoattraction of B cells (44).

IL-6 is induced in decidual tissues at term in labor compared to not in labor (45) and therefore part of parturition. Nevertheless, while increased IL-6 levels were detected in maternal or fetal blood or amniotic fluid, this cytokine is also a well-established marker with an overall good diagnostic accuracy in identifying pregnancies at risk of spontaneous PTB (46–48). This is in agreement with our findings since the patients in our study delivering preterm had enhanced IL-6 level in plasma. Moreover, we found that B cells are substantial contributors to IL-6 release. Even unstimulated B cells from preterm delivering patients released high concentrations of IL-6, stimulation with LPS or CpG alone or together with CD40L did not further enhance the secretion of IL-6. B cells from term-delivering women did not secrete IL-6 without stimulation, but stimulating TD B cells induced IL-6 production. Therefore, B cells contribute to the elevated plasma IL-6 level, significantly associated with preterm delivery (49). Following the culture of isolated B cells for 3 days, we were not able to detect significantly different IL-10 concentrations. We might speculate that after these 3 days, the IL-6-producing B cells overgrew and proliferated much stronger than the IL-10-producing B cells. Since IL-10 was detectable after 5 h and 48 h culturing of PBMCs, we might assume that other immune cells interact with B cells to induce IL-10 and these signals are missing in the 3-day culture of isolated B cells. Moreover, flow cytometry following PMA/ionomycin/Brefeldin A might be more sensitive than the detection of secreted IL-10 in the supernatant after 3 days of culture.

For the direct *ex vivo* measurement of IL-10, we used 5-h incubation with PMA and ionomycin, with or without LPS and CpG. Both TLR agonists were described to induce IL-10 secretion by human Breg cells (14). Despite this, we found that the addition of LPS or CpG does not further increase the Breg population in TD, but slightly enhances Bregs in PTB, resulting in no significant differences between TD and PTB Breg frequencies in these stimulation conditions. We might speculate that B cells from PTB are already activated *in vivo* and thereby respond more strongly toward additional LPS and CpG.

In autoimmune disorders, it was described that B cell-depletion therapy efficiently removes IL-6-producing B cells and thereby ameliorates disorder severity (50). Targeting CD22 and thereby interfering in BCR phosphorylation was shown to reduce B cell-specific IL-6 and TNF-α production, but not IL-10 (51). Although an underlying infection was proven only in one case, undetected infections might at least partly also be present in other PTB cases (8). Leng et al. reported that in chronic chorioamnionitis, decidual B cells secreted high levels of IL-12 and IL-6 (52). Endotoxins like LPS directly stimulate marginal zone B cells via TLR4- and MyD88-pathways for IL-6 production, contributing to systemic inflammatory responses and endotoxic shock (53). IL-6 is an acute-phase protein secreted by several immune cells, including B cells. This lymphokine substantially influences the survival, expansion, and differentiation of B cells and the maintenance of long-lived plasma cells (54). Moreover, IL-6 plays a central role in T cell–B cell responses, since it controls the activity of IL-21 and promotes the commitment of Tfh cells. IL-6 produced by B cells was shown to be necessary and sufficient to induce IL-21 from CD4+ T cells and support the differentiation of activated T cells into Tfh cells (55). This is of interest since IL-6, also IL-21 was strongly enhanced in plasma of our patients undergoing PTB. For B cells, stimulation with IL-21 might result in different outcomes: (1) IL-21 is pro-apoptotic by itself; in the absence of both BCR engagement and T cell help, B cell apoptosis or growth arrest in non-specifically or inappropriately activated B cells would take place (56). (2) In the event of CD40 ligation and either BCR or TLR signaling, IL-21 fundamentally promotes B cell activation and differentiation into memory and plasma cells (57). Moreover, in mice, it was reported that IL-21 stimulation induced IL-10-secreting CD1d^hi^CD5+ Breg cells (58) and human Breg-controlled Tfh cell response (59). Pregnant women have a higher percentage of Tfh cells, which are able to produce IL-6, IL-21, and IL-10 and might contribute to favor humoral immunity during pregnancy (60). Genetic polymorphisms in IL-6, IL-21, and IL-10 are further associated with Recurrent Pregnancy Loss (61, 62). In preeclampsia, the frequency of circulating Tfh cells and the level of IL-21 and IL-6 were higher compared to healthy pregnant women; this is probably associated with the production of disorder-specific autoantibodies (63).

IL-22 is another pro-inflammatory cytokine enhanced in serum of patients undergoing PTB. IL-22 provides a link between infection, B-cell recruitment, and humoral autoimmunity, partly mediated by Th17 cells (64–66). Zhang and colleagues demonstrated that the release of IL-22 correlated with the decrease in CD1d^hi^CD5+ Breg cells, which could efficiently suppress IL-22 (67). Beside IL-6, IL-22, and IL-21, IL-17A is the major cytokine produced by Th17 cells, highly pro-inflammatory T effector cells. Th17 cells might provide B cell help via IL-17 and IL-21, which mediate the differentiation of B cell and class switch recombination (68).

We determined that IL-17A was also enhanced in plasma of women immediately before delivering preterm. Ito et al. (69) demonstrated enhanced IL-17 levels in amniotic fluid of women delivering preterm compared to term labor. In patients with placental insufficiency, the level of IL-17 in serum was higher compared to healthy pregnant women at the same gestational age (70). Progress of healthy pregnancies was also associated with rising IL-17 (70), suggesting that IL-17 might serve as a marker to switch from an anti-inflammatory shape immunity throughout pregnancy toward an inflammatory state necessary to induce labor and birth.

Limitations of our study include the small number of patients and the lack of concrete information about their infection state, as they are often subclinical. Since we could show in this study that the number and function of Breg cells are altered in PTB, we will next recruit a larger cohort of patients. This will enable us to create subgroups to better define the impact of Breg cells within specific etiologies of PTB. Besides, recruitment of patients admitted to hospital with imminent PTB and a follow-up whether PTB will occur or not will further provide information about the exact role of Breg cells in the pathophysiology of PTB. Another possible limitation of our study is that the gestational ages are not matched. However, the imminent labor, not the exact gestational age, is the parameter we employed to compare term and preterm birth. In a follow-up study, we will recruit gestational age-matched, healthy pregnant women to compare their Breg number and plasma cytokine level with PTB patients.

Overall, our data point out that even though an underlying infection was only proven in one case, patients undergoing preterm delivery reflect a pro-inflammatory immune response with a suppression in the generation or maintenance of Breg cells. Besides, the ability of B cells to secrete IL-10 was hampered in PTB patients vs. term-birth patients. Since it is known that Breg cells might also be important for the maintenance of Treg cells, we determined the Treg number in PTB, but found no differences in their frequency. Despite their enormous importance in earlier pregnancy stages (71), their contribution to preterm labor remains to be investigated (72).

We conclude that Breg cells are dysregulated in number and function at the event of preterm birth. We suggest further studies to determine their value as diagnostic tool.

## Data Availability Statement

The datasets generated for this study are available on request to the corresponding author.

## Ethics Statement

The studies involving human participants were reviewed and approved by ethics committee of the Otto-von-Guericke University medical faculty. The patients/participants provided their written informed consent to participate in this study.

## Author Contributions

MB collected patient samples, designed and carried out experiments, analyzed the data, and wrote the manuscript. K-NC performed experiments. AR, AO, and S-DC recruited patients. RH supported the flow cytometry of patients' samples. AZ developed the working hypothesis, designed experiments, supervised the work, wrote the manuscript, and provided the financial support. All authors reviewed the manuscript.

### Conflict of Interest

The authors declare that the research was conducted in the absence of any commercial or financial relationships that could be construed as a potential conflict of interest.
