# Supplementary material for: Regulatory B Cells Are Decreased and Impaired in Their Function in Peripheral Maternal Blood in Pre-term Birth
Source: Front Immunol. 2020 Mar 20;11:386. doi: 10.3389/fimmu.2020.00386 (PMC7099879; doi:10.3389/fimmu.2020.00386)
Supplement: Supplementary file 1 [file Presentation_1.PPTX]

## Slide 1
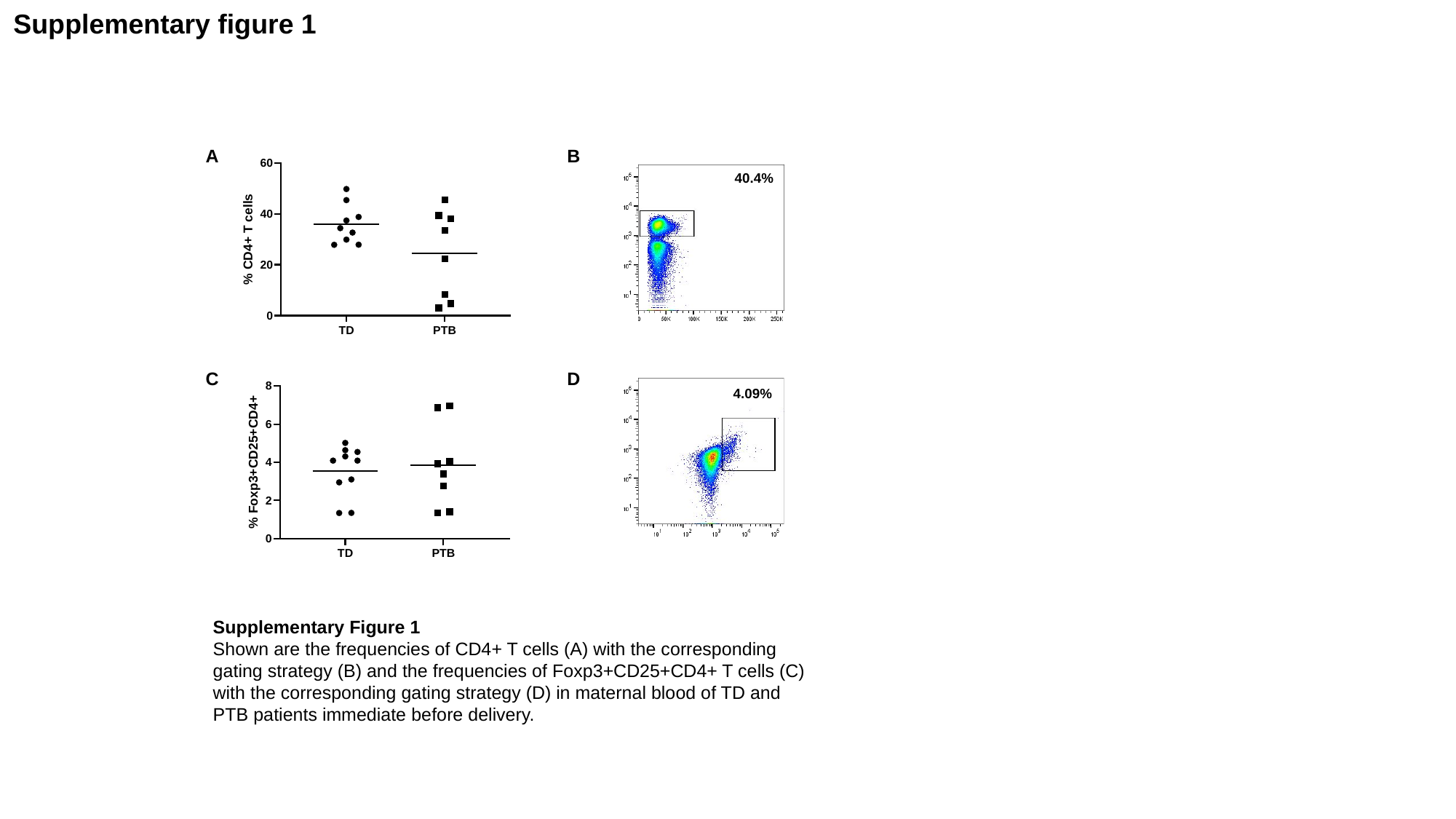

Supplementary figure 1
A
B
40.4%
C
D
4.09%
Supplementary Figure 1
Shown are the frequencies of CD4+ T cells (A) with the corresponding gating strategy (B) and the frequencies of Foxp3+CD25+CD4+ T cells (C) with the corresponding gating strategy (D) in maternal blood of TD and PTB patients immediate before delivery.
