# Supplementary material for: Regulatory B Cells Are Decreased and Impaired in Their Function in Peripheral Maternal Blood in Pre-term Birth
Source: Front Immunol. 2020 Mar 20;11:386. doi: 10.3389/fimmu.2020.00386 (PMC7099879; doi:10.3389/fimmu.2020.00386)
Supplement: Supplementary file 2 [file Presentation_2.PPTX]

## Slide 1
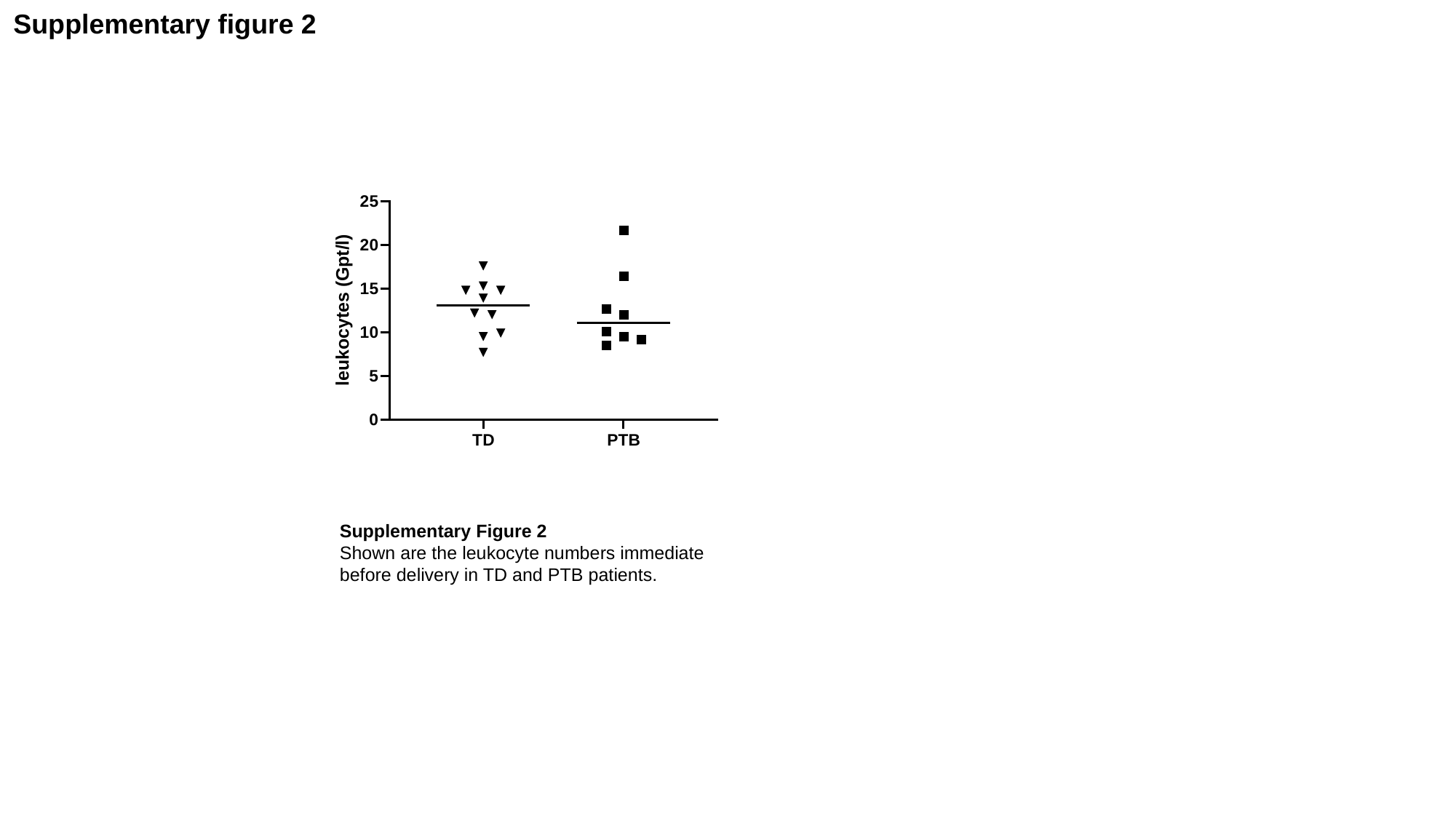

Supplementary figure 2
Supplementary Figure 2
Shown are the leukocyte numbers immediate before delivery in TD and PTB patients.
