# Supplementary material for: Regulatory B Cells Are Decreased and Impaired in Their Function in Peripheral Maternal Blood in Pre-term Birth
Source: Front Immunol. 2020 Mar 20;11:386. doi: 10.3389/fimmu.2020.00386 (PMC7099879; doi:10.3389/fimmu.2020.00386)
Supplement: Supplementary file 3 [file Presentation_3.PPTX]

## Slide 1
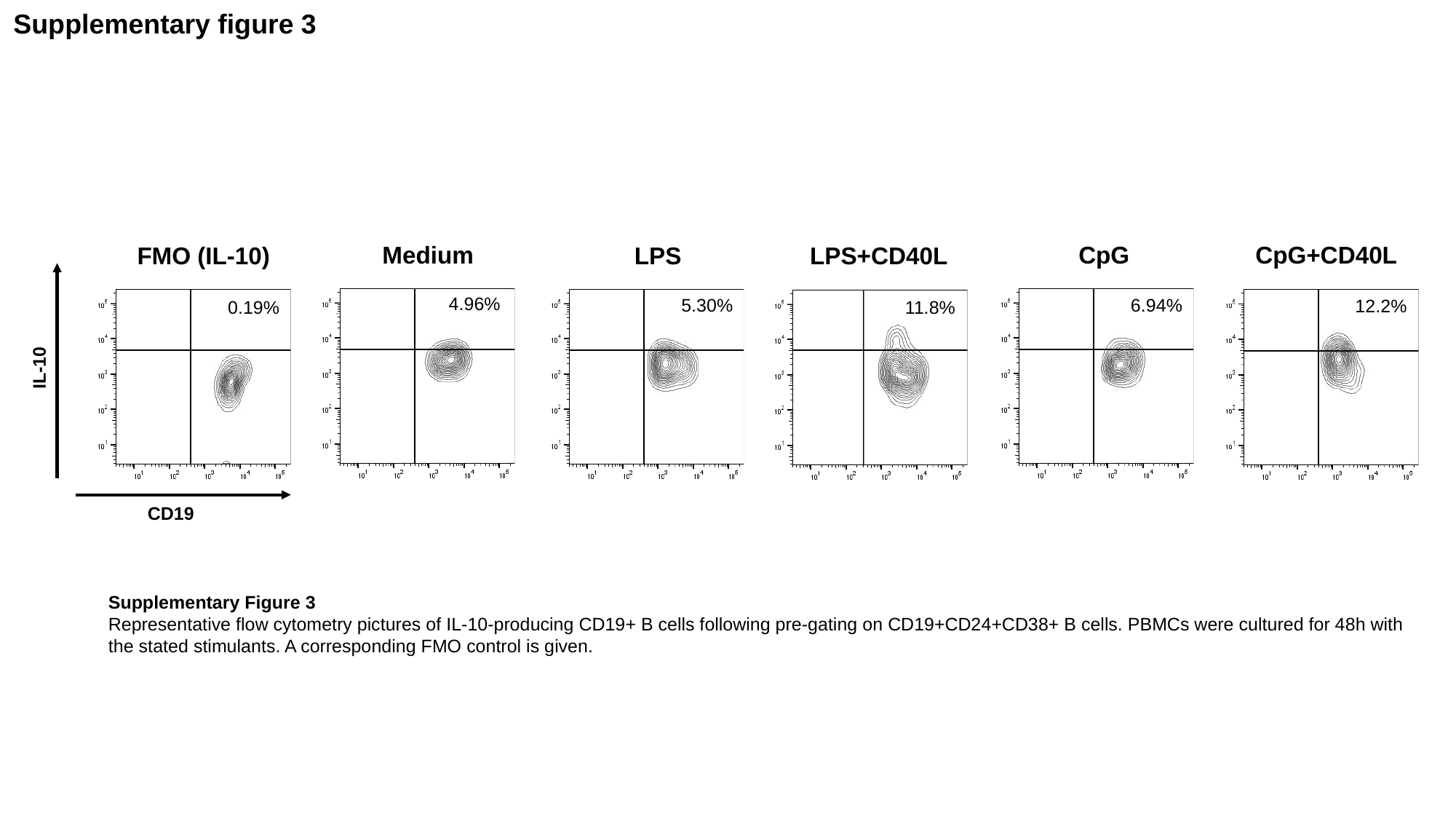

Supplementary figure 3
Medium
4.96%
CpG
6.94%
CpG+CD40L
12.2%
FMO (IL-10)
0.19%
LPS
5.30%
LPS+CD40L
11.8%
IL-10
CD19
Supplementary Figure 3
Representative flow cytometry pictures of IL-10-producing CD19+ B cells following pre-gating on CD19+CD24+CD38+ B cells. PBMCs were cultured for 48h with the stated stimulants. A corresponding FMO control is given.
