# Supplementary material for: Regulatory B Cells Are Decreased and Impaired in Their Function in Peripheral Maternal Blood in Pre-term Birth
Source: Front Immunol. 2020 Mar 20;11:386. doi: 10.3389/fimmu.2020.00386 (PMC7099879; doi:10.3389/fimmu.2020.00386)
Supplement: Supplementary file 4 [file Presentation_4.PPTX]

## Slide 1
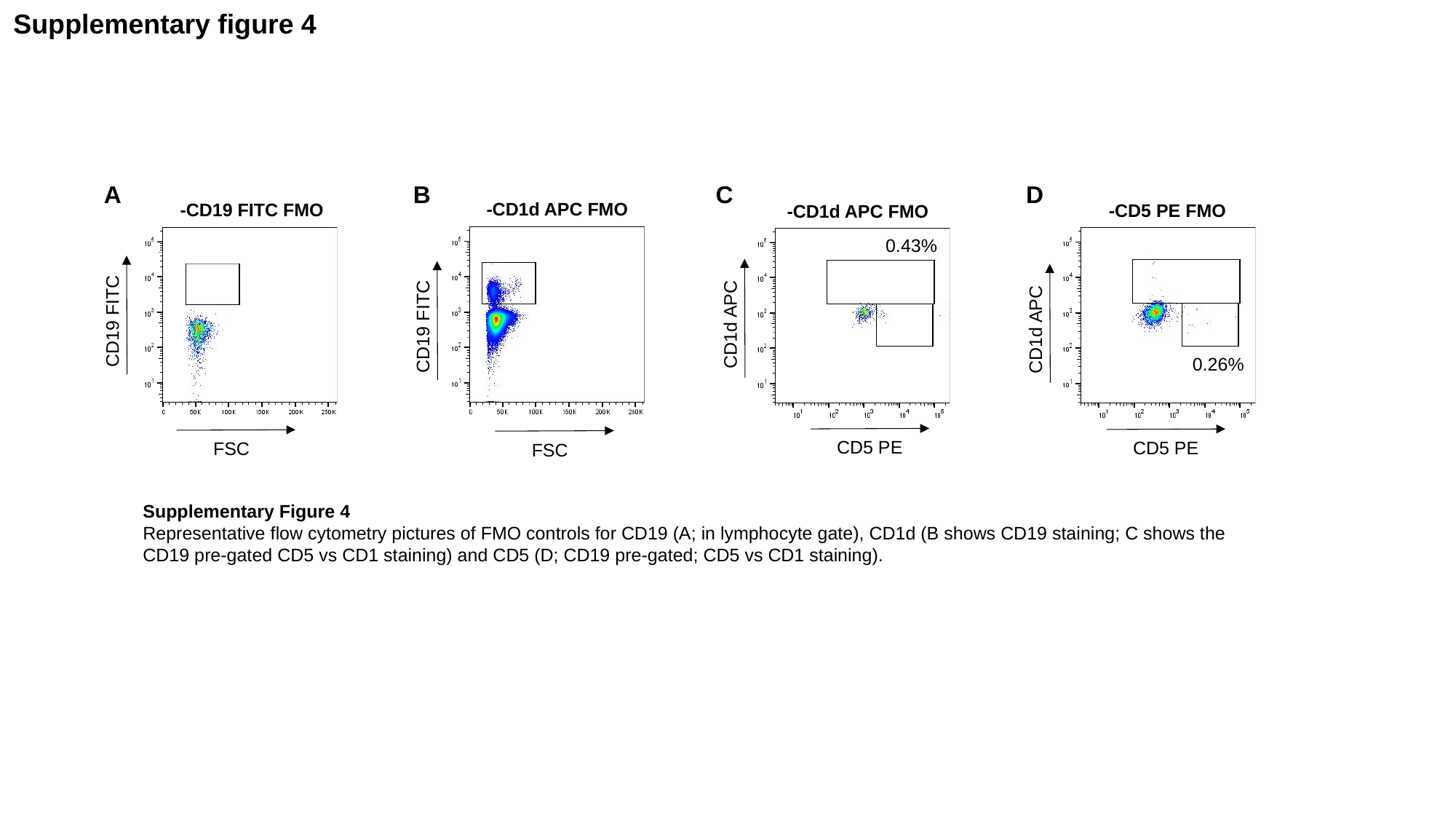

Supplementary figure 4
A
B
C
D
-CD1d APC FMO
CD19 FITC
FSC
-CD19 FITC FMO
CD19 FITC
FSC
-CD5 PE FMO
CD1d APC
0.26%
CD5 PE
-CD1d APC FMO
0.43%
CD1d APC
CD5 PE
Supplementary Figure 4
Representative flow cytometry pictures of FMO controls for CD19 (A; in lymphocyte gate), CD1d (B shows CD19 staining; C shows the CD19 pre-gated CD5 vs CD1 staining) and CD5 (D; CD19 pre-gated; CD5 vs CD1 staining).
