# Supplementary material for: Regulatory B Cells Are Decreased and Impaired in Their Function in Peripheral Maternal Blood in Pre-term Birth
Source: Front Immunol. 2020 Mar 20;11:386. doi: 10.3389/fimmu.2020.00386 (PMC7099879; doi:10.3389/fimmu.2020.00386)
Supplement: Supplementary file 5 [file Presentation_5.PPTX]

## Slide 1
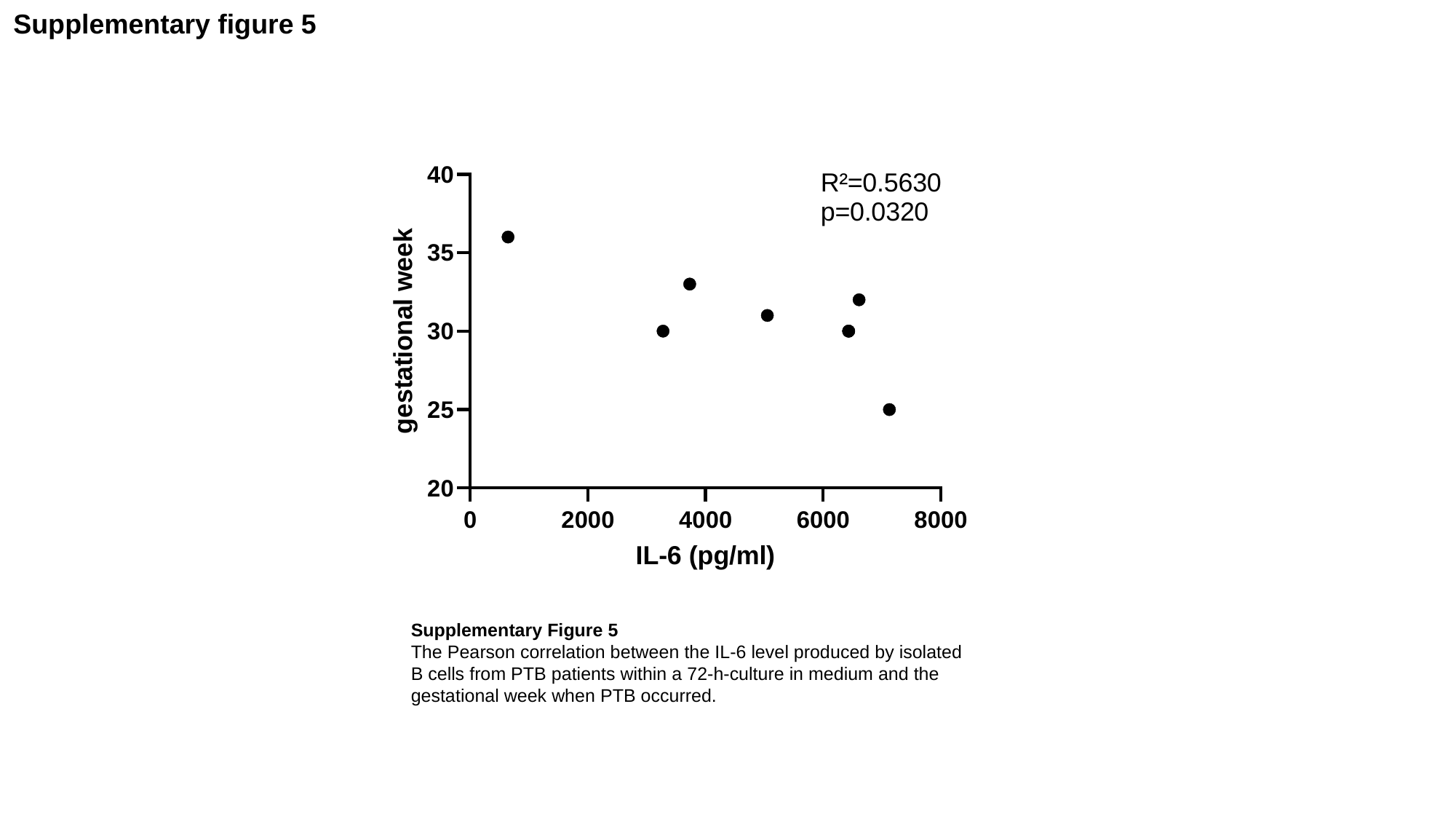

Supplementary figure 5
Supplementary Figure 5
The Pearson correlation between the IL-6 level produced by isolated B cells from PTB patients within a 72-h-culture in medium and the gestational week when PTB occurred.
