# Supplementary material for: Regulatory B Cells Are Decreased and Impaired in Their Function in Peripheral Maternal Blood in Pre-term Birth
Source: Front Immunol. 2020 Mar 20;11:386. doi: 10.3389/fimmu.2020.00386 (PMC7099879; doi:10.3389/fimmu.2020.00386)
Supplement: Supplementary file 6 [file Presentation_6.PPTX]

## Slide 1
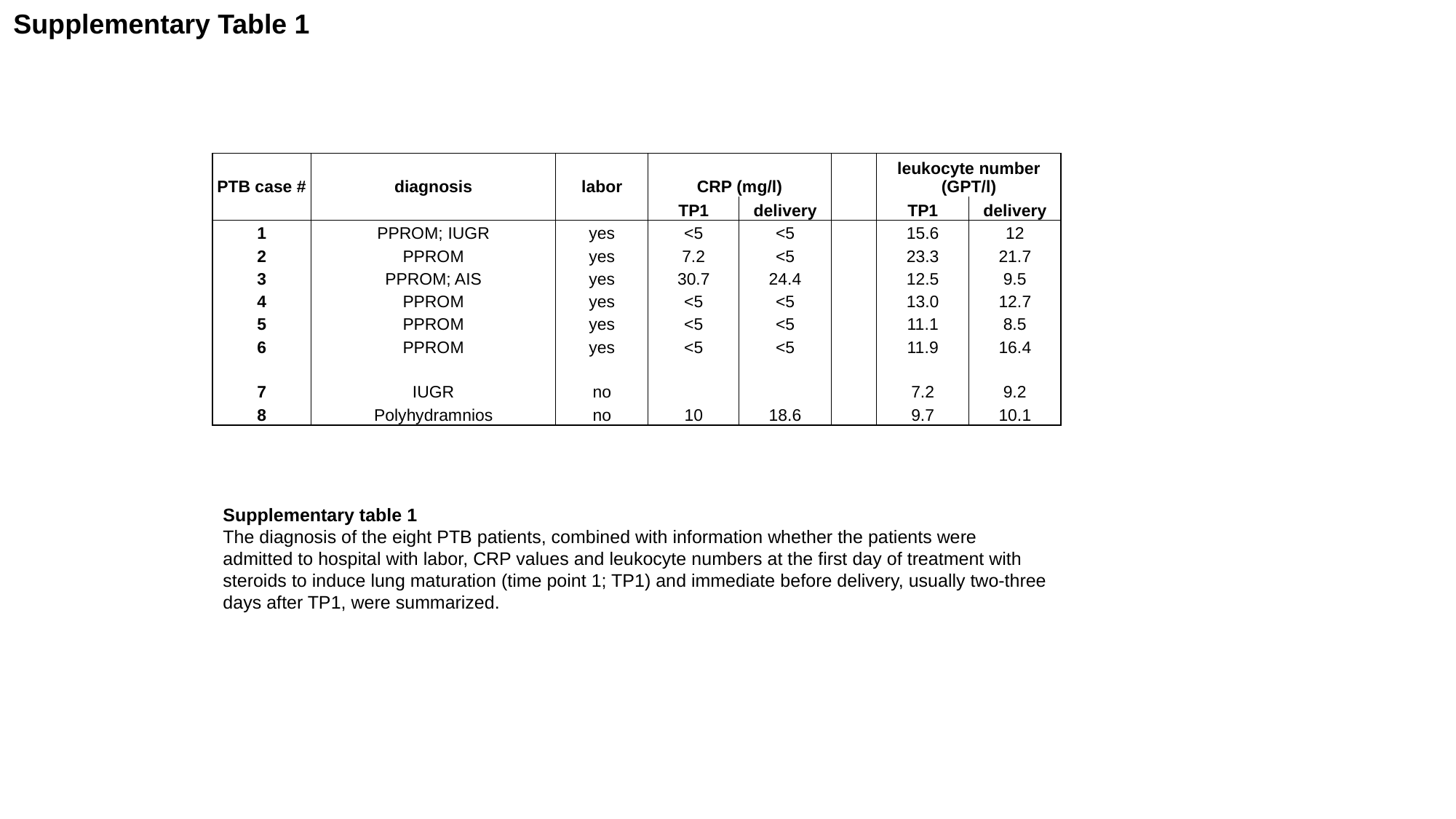

Supplementary Table 1
| PTB case # | diagnosis | labor | CRP (mg/l) | | | leukocyte number (GPT/l) | |
| --- | --- | --- | --- | --- | --- | --- | --- |
| | | | TP1 | delivery | | TP1 | delivery |
| 1 | PPROM; IUGR | yes | <5 | <5 | | 15.6 | 12 |
| 2 | PPROM | yes | 7.2 | <5 | | 23.3 | 21.7 |
| 3 | PPROM; AIS | yes | 30.7 | 24.4 | | 12.5 | 9.5 |
| 4 | PPROM | yes | <5 | <5 | | 13.0 | 12.7 |
| 5 | PPROM | yes | <5 | <5 | | 11.1 | 8.5 |
| 6 | PPROM | yes | <5 | <5 | | 11.9 | 16.4 |
| | | | | | | | |
| 7 | IUGR | no | | | | 7.2 | 9.2 |
| 8 | Polyhydramnios | no | 10 | 18.6 | | 9.7 | 10.1 |
Supplementary table 1
The diagnosis of the eight PTB patients, combined with information whether the patients were admitted to hospital with labor, CRP values and leukocyte numbers at the first day of treatment with steroids to induce lung maturation (time point 1; TP1) and immediate before delivery, usually two-three days after TP1, were summarized.
